# 7-Chloro-4-[(*E*)-2-(4-methoxy­benzyl­idene)hydrazin-1-yl]quinoline monohydrate

**DOI:** 10.1107/S1600536810006598

**Published:** 2010-02-27

**Authors:** Marcelle de Lima Ferreira, Marcus V. N. de Souza, R. Alan Howie, Edward R. T. Tiekink, James L. Wardell, Solange M. S. V. Wardell

**Affiliations:** a Instituto de Tecnologia em Farmacos, Fundação Oswaldo Cruz (FIOCRUZ), FarManguinhos, Rua Sizenando Nabuco, 100, Manguinhos, 21041-250 Rio de Janeiro, RJ, Brazil; b Department of Chemistry, University of Aberdeen, Old Aberdeen AB15 5NY, Scotland; c Department of Chemistry, University of Malaya, 50603 Kuala Lumpur, Malaysia; d Centro de Desenvolvimento Tecnológico em Saúde (CDTS), Fundação Oswaldo Cruz (FIOCRUZ), Casa Amarela, Campus de Manguinhos, Av. Brasil 4365, 21040-900 Rio de Janeiro, RJ, Brazil; e CHEMSOL, 1 Harcourt Road, Aberdeen AB15 5NY, Scotland

## Abstract

The organic mol­ecule in the title hydrate, C_17_H_14_ClN_3_O·H_2_O, has a small but significant twist from planarity, as seen in the dihedral angle of 12.10 (17)° between the quinoline and benzene rings. The conformation about the C=N bond is *E*. Chains along the *b* axis are formed in the crystal structure aided by water–quinoline O—H⋯N (× 2) and hydrazone–water N—H⋯O hydrogen bonds. Layers of these chains stack along the *a* axis *via* C—H⋯π and π–π inter­actions [ring centroid–ring centroid distance = 3.674 (2) Å]. C—H⋯O inter­actions are also present.

## Related literature

For background to the pharmacological activity of quinoline derivatives, see: Warshakoon *et al.* (2006 ▶). For recent studies into quinoline-based anti-malarials, see: Andrade *et al.* (2007 ▶); de Souza *et al.* (2005 ▶). For related structures, see: Kaiser *et al.* (2009 ▶); de Souza *et al.* (2009 ▶, 2010 ▶).
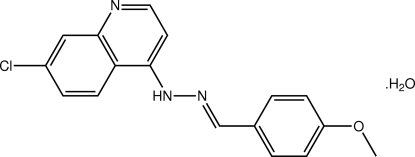


## Experimental

### 

#### Crystal data

C_17_H_14_ClN_3_O·H_2_O
*M* *_r_* = 329.78
Triclinic, 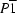

*a* = 7.0086 (6) Å
*b* = 9.2384 (8) Å
*c* = 13.3701 (12) Å
α = 100.026 (4)°
β = 103.903 (5)°
γ = 107.000 (5)°
*V* = 775.27 (12) Å^3^
*Z* = 2
Mo *K*α radiation
μ = 0.26 mm^−1^
*T* = 120 K
0.12 × 0.04 × 0.02 mm

#### Data collection

Nonius KappaCCD area-detector diffractometer
Absorption correction: multi-scan (*SADABS*; Sheldrick, 2007 ▶) *T*_min_ = 0.686, *T*_max_ = 1.000
10514 measured reflections
2702 independent reflections
2037 reflections with *I* > 2σ(*I*)
*R*_int_ = 0.064

#### Refinement

*R*[*F* ^2^ > 2σ(*F* ^2^)] = 0.069
*wR*(*F* ^2^) = 0.150
*S* = 1.02
2702 reflections
215 parameters
H atoms treated by a mixture of independent and constrained refinement
Δρ_max_ = 0.30 e Å^−3^
Δρ_min_ = −0.36 e Å^−3^

### 

Data collection: *COLLECT* (Hooft, 1998 ▶); cell refinement: *DENZO* (Otwinowski & Minor, 1997 ▶) and *COLLECT*; data reduction: *DENZO* and *COLLECT*; program(s) used to solve structure: *SHELXS97* (Sheldrick, 2008 ▶); program(s) used to refine structure: *SHELXL97* (Sheldrick, 2008 ▶); molecular graphics: *ORTEP-3* (Farrugia, 1997 ▶) and *DIAMOND* (Brandenburg, 2006 ▶); software used to prepare material for publication: *publCIF* (Westrip, 2010 ▶).

## Supplementary Material

Crystal structure: contains datablocks global, I. DOI: 10.1107/S1600536810006598/lh5002sup1.cif

Structure factors: contains datablocks I. DOI: 10.1107/S1600536810006598/lh5002Isup2.hkl

Additional supplementary materials:  crystallographic information; 3D view; checkCIF report

## Figures and Tables

**Table 1:** Hydrogen-bond geometry (Å, °) *Cg* is the centroid of the C11–C16 ring.

*D*—H⋯*A*	*D*—H	H⋯*A*	*D*⋯*A*	*D*—H⋯*A*
O1w—H1w⋯N1^i^	0.85 (5)	2.02 (5)	2.867 (4)	172 (5)
O1w—H2w⋯N1	0.85 (5)	2.20 (5)	3.047 (5)	175 (5)
N2—H2n⋯O1w^ii^	0.88	2.18	3.007 (4)	157
C5—H5⋯O1w^ii^	0.95	2.45	3.380 (5)	165
C17—H17a⋯*Cg*^iii^	0.98	2.65	3.508 (5)	147

Symmetry codes: (i) 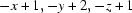; (ii) 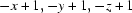; (iii) 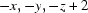.

**Fig. 1.**
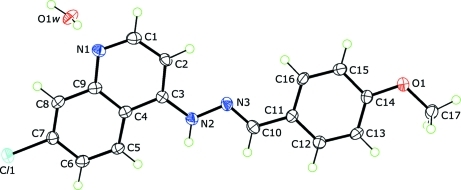
The molecular structure of the components comprising the asymmetric unit in (I) showing the atom-labelling scheme and displacement ellipsoids at the 50% probability level.

**Fig. 2.**
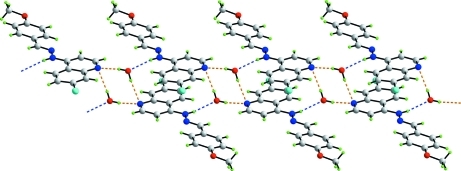
View of the supramolecular chain in (I) showing the O–H···N and N–H···O hydrogen bonding as orange and blue dashed lines, respectively. The C–H···O contacts are represented as green dashed lines. Colour code: Cl, cyan; O, red; N, blue; C, grey; and H, green.

**Fig. 3.**
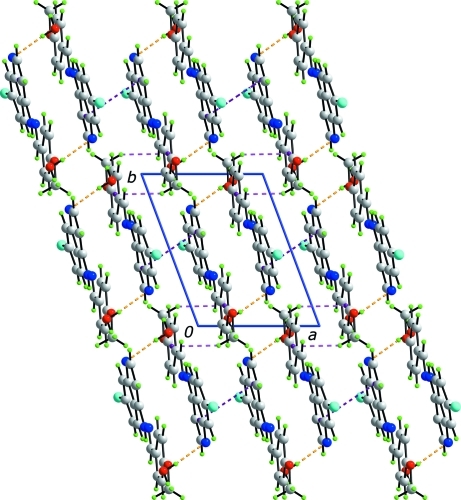
A view of the stacking of layers in (I); O–H···N hydrogen bonding is shown as orange dashed lines. The layers are linked by π–π (purple dashed lines) and C–H···π contacts (pink dashed lines). Colour code: Cl, cyan; O, red; N, blue; C, grey; and H, green.

## supplementary crystallographic information

### Comment

Quinoline derivatives display biological activity (Warshakoon *et al.*, 2006) and in this context attract interest as potential anti-malarial agents (Andrade *et al.* 2007; de Souza *et al.*,  2005). Complementing biological studies are structural investigations (Kaiser *et al.*, 2009; de Souza *et al.*, 2009; de Souza *et al.*, 2010), and the crystal structure of the title hydrate, (I), was investigated as a part of these on-going studies.

The molecular structure of the organic component of (I), Fig. 1, shows a small twist from planarity with the dihedral angle formed between the quinoline (maximum deviation = 0.039 (4) Å for the C6 atom) and benzene rings being 12.10 (17) °. The major deviation of a torsion angle from 0 or 180 ° is found in the C3–N2–N3–C10 torsion angle of 172.4 (3) °. The conformation about the C10═N3 bond [1.274 (5) Å] is *E*. The crystal packing is stabilised by a variety of hydrogen bonding interactions, Table 1. The water molecule accepts a hydrogen bond from the hydrazone-N2 atom and forms donor interactions with symmetry related quinoline-N1 atoms, the latter leading to eight-membered {···OHO···N}_2_ synthons. The resulting supramolecular chain along the *b* axis, Fig. 2, is reinforced by a C–H···O contact, Table 1. The chains are arranged in layers in the *bc* plane with the most significant interactions between the layers being of the type π–π with the closest of these occurring between centrosymmetrically related N1,C1—C4,C9 rings [ring centroid(N1,C1—C4,C9)···ring centroid(N1,C1—C4,C9)^i^ distance = 3.674 (2) Å for *i*: -*x*, 1-*y*, 1-*z*], Fig. 3. In addition to these interactions, C–H···π contacts also occur between layers, Table 1.

### Experimental

A solution of 7-chloro-4-quinolinylhydrazine (0.2 g, 1.03 mmol) and 4-methoxybenzaldehyde (1.24 mmol) in ethanol (5 ml) was stirred at room temperature until TLC indicated complete consumption of the hydrazine. The reaction mixture was rotary evaporated, the residue washed well with cold Et_2_O (3 x 10 ml), and recrystallised from moist ethanol, yield 85%, m.pt. 417–418 K. IR [KBr, cm^-1^] ν: 3120 (NH), 1565(N═C). MS/ESI: *m*/*z* [M—H]^+^: 310.8.

### Refinement

The N- and C-bound H atoms were geometrically placed (N–H = 0.88 Å and C–H = 0.95–0.98 Å) and refined as riding with *U*_iso_(H) = 1.2–1.5*U*_eq_(C,*N*). The water-bound H atoms were located from a difference map and refined with *U*_iso_(H) = 1.5*U*_eq_(O).

### Crystal data

**Table:**

C_17_H_14_ClN_3_O·H_2_O	*Z* = 2
*M**_r_* = 329.78	*F*(000) = 344
Triclinic, *P*1	*D*_x_ = 1.413 Mg m^−^^3^
Hall symbol: -P 1	Mo *K*α radiation, λ = 0.71073 Å
*a* = 7.0086 (6) Å	Cell parameters from 27436 reflections
*b* = 9.2384 (8) Å	θ = 2.9–27.5°
*c* = 13.3701 (12) Å	µ = 0.26 mm^−^^1^
α = 100.026 (4)°	*T* = 120 K
β = 103.903 (5)°	Needle, colourless
γ = 107.000 (5)°	0.12 × 0.04 × 0.02 mm
*V* = 775.27 (12) Å^3^	

### Data collection

**Table:**

Nonius KappaCCD area-detector diffractometer	2702 independent reflections
Radiation source: Enraf Nonius FR591 rotating anode	2037 reflections with *I* > 2σ(*I*)
10 cm confocal mirrors	*R*_int_ = 0.064
Detector resolution: 9.091 pixels mm^-1^	θ_max_ = 25.0°, θ_min_ = 3.1°
φ and ω scans	*h* = −8→8
Absorption correction: multi-scan (*SADABS*; Sheldrick, 2007)	*k* = −10→10
*T*_min_ = 0.686, *T*_max_ = 1.000	*l* = −15→15
10514 measured reflections	

### Refinement

**Table:**

Refinement on *F*^2^	Primary atom site location: structure-invariant direct methods
Least-squares matrix: full	Secondary atom site location: difference Fourier map
*R*[*F*^2^ > 2σ(*F*^2^)] = 0.069	Hydrogen site location: inferred from neighbouring sites
*wR*(*F*^2^) = 0.150	H atoms treated by a mixture of independent and constrained refinement
*S* = 1.02	*w* = 1/[σ^2^(*F*_o_^2^) + (0.0109*P*)^2^ + 2.93*P*] where *P* = (*F*_o_^2^ + 2*F*_c_^2^)/3
2702 reflections	(Δ/σ)_max_ = 0.001
215 parameters	Δρ_max_ = 0.30 e Å^−^^3^
0 restraints	Δρ_min_ = −0.36 e Å^−^^3^

### Special details

**Table:**

Geometry. All s.u.'s (except the s.u. in the dihedral angle between two l.s. planes) are estimated using the full covariance matrix. The cell s.u.'s are taken into account individually in the estimation of s.u.'s in distances, angles and torsion angles; correlations between s.u.'s in cell parameters are only used when they are defined by crystal symmetry. An approximate (isotropic) treatment of cell s.u.'s is used for estimating s.u.'s involving l.s. planes.
Refinement. Refinement of *F*^2^ against ALL reflections. The weighted *R*-factor wR and goodness of fit *S* are based on *F*^2^, conventional *R*-factors *R* are based on *F*, with *F* set to zero for negative *F*^2^. The threshold expression of *F*^2^ > 2σ(*F*^2^) is used only for calculating *R*-factors(gt) etc. and is not relevant to the choice of reflections for refinement. *R*-factors based on *F*^2^ are statistically about twice as large as those based on *F*, and *R*- factors based on ALL data will be even larger.

### Fractional atomic coordinates and isotropic or equivalent isotropic displacement parameters (Å^2^)

**Table:**

	*x*	*y*	*z*	*U*_iso_*/*U*_eq_	
Cl1	0.12492 (19)	0.53097 (12)	0.13214 (8)	0.0337 (3)	
O1	0.3226 (4)	0.0839 (3)	1.1296 (2)	0.0268 (7)	
N1	0.2963 (5)	0.7665 (4)	0.5289 (2)	0.0233 (7)	
N2	0.2484 (5)	0.3358 (4)	0.6035 (2)	0.0226 (7)	
H2N	0.2193	0.2506	0.5527	0.027*	
N3	0.2774 (5)	0.3291 (4)	0.7081 (2)	0.0220 (7)	
C1	0.3251 (6)	0.7500 (4)	0.6279 (3)	0.0247 (9)	
H1	0.3611	0.8418	0.6835	0.030*	
C2	0.3074 (6)	0.6112 (4)	0.6569 (3)	0.0217 (9)	
H2	0.3239	0.6092	0.7292	0.026*	
C3	0.2653 (6)	0.4750 (4)	0.5796 (3)	0.0178 (8)	
C4	0.2376 (6)	0.4842 (4)	0.4712 (3)	0.0188 (8)	
C5	0.2011 (6)	0.3562 (4)	0.3853 (3)	0.0203 (8)	
H5	0.1959	0.2576	0.3985	0.024*	
C6	0.1728 (6)	0.3715 (4)	0.2828 (3)	0.0215 (8)	
H6	0.1518	0.2852	0.2259	0.026*	
C7	0.1753 (6)	0.5170 (4)	0.2634 (3)	0.0211 (8)	
C8	0.2147 (6)	0.6443 (4)	0.3441 (3)	0.0225 (9)	
H8	0.2180	0.7417	0.3292	0.027*	
C9	0.2507 (6)	0.6313 (4)	0.4502 (3)	0.0201 (8)	
C10	0.2358 (6)	0.1919 (5)	0.7224 (3)	0.0235 (9)	
H10	0.1879	0.1044	0.6620	0.028*	
C11	0.2591 (6)	0.1642 (4)	0.8281 (3)	0.0199 (8)	
C12	0.2046 (6)	0.0122 (4)	0.8385 (3)	0.0222 (9)	
H12	0.1524	−0.0724	0.7759	0.027*	
C13	0.2235 (6)	−0.0212 (4)	0.9368 (3)	0.0214 (8)	
H13	0.1854	−0.1267	0.9415	0.026*	
C14	0.2985 (6)	0.1016 (4)	1.0276 (3)	0.0217 (8)	
C15	0.3569 (6)	0.2567 (4)	1.0201 (3)	0.0229 (9)	
H15	0.4099	0.3408	1.0830	0.027*	
C16	0.3379 (6)	0.2878 (4)	0.9220 (3)	0.0227 (9)	
H16	0.3781	0.3934	0.9176	0.027*	
C17	0.2580 (7)	−0.0735 (4)	1.1403 (3)	0.0268 (9)	
H17A	0.1093	−0.1280	1.0997	0.040*	
H17B	0.2787	−0.0709	1.2158	0.040*	
H17C	0.3418	−0.1291	1.1127	0.040*	
O1W	0.7337 (5)	0.9550 (3)	0.5298 (2)	0.0309 (7)	
H1W	0.736 (8)	1.039 (6)	0.511 (4)	0.046*	
H2W	0.610 (8)	0.908 (6)	0.530 (4)	0.046*	

### Atomic displacement parameters (Å^2^)

**Table:**

	*U*^11^	*U*^22^	*U*^33^	*U*^12^	*U*^13^	*U*^23^
Cl1	0.0524 (7)	0.0299 (6)	0.0200 (5)	0.0158 (5)	0.0098 (5)	0.0088 (4)
O1	0.0346 (17)	0.0247 (15)	0.0202 (14)	0.0082 (13)	0.0077 (12)	0.0084 (11)
N1	0.0272 (19)	0.0234 (17)	0.0200 (17)	0.0102 (15)	0.0088 (14)	0.0031 (13)
N2	0.0285 (19)	0.0215 (17)	0.0191 (17)	0.0116 (15)	0.0062 (14)	0.0047 (13)
N3	0.0258 (19)	0.0246 (18)	0.0207 (17)	0.0122 (15)	0.0103 (14)	0.0084 (14)
C1	0.025 (2)	0.021 (2)	0.026 (2)	0.0067 (17)	0.0097 (18)	0.0019 (16)
C2	0.025 (2)	0.024 (2)	0.0171 (19)	0.0085 (17)	0.0100 (17)	0.0035 (16)
C3	0.0147 (19)	0.0195 (19)	0.0205 (19)	0.0066 (15)	0.0067 (16)	0.0058 (15)
C4	0.0126 (19)	0.0185 (19)	0.024 (2)	0.0037 (15)	0.0070 (16)	0.0050 (15)
C5	0.020 (2)	0.0164 (19)	0.024 (2)	0.0070 (16)	0.0057 (16)	0.0047 (15)
C6	0.018 (2)	0.020 (2)	0.025 (2)	0.0038 (16)	0.0088 (16)	0.0041 (16)
C7	0.021 (2)	0.023 (2)	0.021 (2)	0.0075 (17)	0.0077 (16)	0.0073 (16)
C8	0.024 (2)	0.0175 (19)	0.027 (2)	0.0078 (16)	0.0077 (17)	0.0071 (16)
C9	0.016 (2)	0.0194 (19)	0.024 (2)	0.0046 (16)	0.0096 (16)	0.0033 (16)
C10	0.024 (2)	0.028 (2)	0.020 (2)	0.0108 (17)	0.0086 (17)	0.0053 (16)
C11	0.018 (2)	0.0199 (19)	0.023 (2)	0.0068 (16)	0.0085 (16)	0.0071 (16)
C12	0.024 (2)	0.022 (2)	0.023 (2)	0.0102 (17)	0.0098 (17)	0.0062 (16)
C13	0.023 (2)	0.0154 (19)	0.028 (2)	0.0066 (16)	0.0109 (17)	0.0061 (16)
C14	0.022 (2)	0.023 (2)	0.022 (2)	0.0076 (17)	0.0093 (17)	0.0085 (16)
C15	0.022 (2)	0.019 (2)	0.024 (2)	0.0066 (17)	0.0041 (17)	0.0036 (16)
C16	0.024 (2)	0.0168 (19)	0.026 (2)	0.0044 (16)	0.0073 (17)	0.0093 (16)
C17	0.032 (2)	0.025 (2)	0.028 (2)	0.0113 (18)	0.0109 (18)	0.0114 (17)
O1W	0.0347 (18)	0.0205 (15)	0.0434 (18)	0.0117 (14)	0.0171 (15)	0.0123 (13)

### Geometric parameters (Å, °)

**Table:**

Cl1—C7	1.740 (4)	C7—C8	1.360 (5)
O1—C14	1.377 (4)	C8—C9	1.413 (5)
O1—C17	1.435 (5)	C8—H8	0.9500
N1—C1	1.332 (5)	C10—C11	1.458 (5)
N1—C9	1.385 (5)	C10—H10	0.9500
N2—C3	1.357 (5)	C11—C12	1.385 (5)
N2—N3	1.380 (4)	C11—C16	1.409 (5)
N2—H2N	0.8800	C12—C13	1.386 (5)
N3—C10	1.274 (5)	C12—H12	0.9500
C1—C2	1.383 (5)	C13—C14	1.379 (5)
C1—H1	0.9500	C13—H13	0.9500
C2—C3	1.386 (5)	C14—C15	1.400 (5)
C2—H2	0.9500	C15—C16	1.374 (5)
C3—C4	1.435 (5)	C15—H15	0.9500
C4—C9	1.417 (5)	C16—H16	0.9500
C4—C5	1.411 (5)	C17—H17A	0.9800
C5—C6	1.374 (5)	C17—H17B	0.9800
C5—H5	0.9500	C17—H17C	0.9800
C6—C7	1.409 (5)	O1W—H1W	0.85 (5)
C6—H6	0.9500	O1W—H2W	0.85 (5)
			
C14—O1—C17	117.0 (3)	N1—C9—C8	116.8 (3)
C1—N1—C9	115.5 (3)	C4—C9—C8	119.7 (3)
C3—N2—N3	119.4 (3)	N3—C10—C11	122.5 (3)
C3—N2—H2N	120.3	N3—C10—H10	118.8
N3—N2—H2N	120.3	C11—C10—H10	118.8
C10—N3—N2	115.5 (3)	C12—C11—C16	117.8 (3)
N1—C1—C2	125.9 (4)	C12—C11—C10	119.9 (3)
N1—C1—H1	117.0	C16—C11—C10	122.3 (3)
C2—C1—H1	117.0	C11—C12—C13	122.5 (4)
C1—C2—C3	119.5 (3)	C11—C12—H12	118.8
C1—C2—H2	120.3	C13—C12—H12	118.8
C3—C2—H2	120.3	C14—C13—C12	118.8 (3)
N2—C3—C2	122.1 (3)	C14—C13—H13	120.6
N2—C3—C4	120.0 (3)	C12—C13—H13	120.6
C2—C3—C4	117.9 (3)	O1—C14—C13	124.4 (3)
C9—C4—C5	118.4 (3)	O1—C14—C15	115.4 (3)
C9—C4—C3	117.7 (3)	C13—C14—C15	120.2 (3)
C5—C4—C3	123.9 (3)	C16—C15—C14	120.2 (4)
C6—C5—C4	121.3 (3)	C16—C15—H15	119.9
C6—C5—H5	119.3	C14—C15—H15	119.9
C4—C5—H5	119.3	C15—C16—C11	120.5 (3)
C5—C6—C7	119.0 (3)	C15—C16—H16	119.8
C5—C6—H6	120.5	C11—C16—H16	119.8
C7—C6—H6	120.5	O1—C17—H17A	109.5
C8—C7—C6	121.6 (3)	O1—C17—H17B	109.5
C8—C7—Cl1	120.0 (3)	H17A—C17—H17B	109.5
C6—C7—Cl1	118.3 (3)	O1—C17—H17C	109.5
C7—C8—C9	119.8 (3)	H17A—C17—H17C	109.5
C7—C8—H8	120.1	H17B—C17—H17C	109.5
C9—C8—H8	120.1	H1W—O1W—H2W	108 (5)
N1—C9—C4	123.5 (3)		
			
C3—N2—N3—C10	172.4 (3)	C3—C4—C9—N1	2.5 (5)
C9—N1—C1—C2	−2.0 (6)	C5—C4—C9—C8	3.8 (5)
N1—C1—C2—C3	3.1 (6)	C3—C4—C9—C8	−177.0 (3)
N3—N2—C3—C2	−1.1 (5)	C7—C8—C9—N1	178.1 (3)
N3—N2—C3—C4	179.4 (3)	C7—C8—C9—C4	−2.4 (6)
C1—C2—C3—N2	179.3 (3)	N2—N3—C10—C11	179.7 (3)
C1—C2—C3—C4	−1.2 (5)	N3—C10—C11—C12	177.7 (4)
N2—C3—C4—C9	178.2 (3)	N3—C10—C11—C16	−2.8 (6)
C2—C3—C4—C9	−1.3 (5)	C16—C11—C12—C13	0.5 (6)
N2—C3—C4—C5	−2.7 (5)	C10—C11—C12—C13	180.0 (4)
C2—C3—C4—C5	177.8 (3)	C11—C12—C13—C14	0.3 (6)
C9—C4—C5—C6	−1.8 (5)	C17—O1—C14—C13	−2.2 (5)
C3—C4—C5—C6	179.1 (4)	C17—O1—C14—C15	178.0 (3)
C4—C5—C6—C7	−1.6 (6)	C12—C13—C14—O1	179.4 (4)
C5—C6—C7—C8	3.1 (6)	C12—C13—C14—C15	−0.9 (6)
C5—C6—C7—Cl1	−176.5 (3)	O1—C14—C15—C16	−179.5 (3)
C6—C7—C8—C9	−1.1 (6)	C13—C14—C15—C16	0.7 (6)
Cl1—C7—C8—C9	178.5 (3)	C14—C15—C16—C11	0.1 (6)
C1—N1—C9—C4	−0.9 (5)	C12—C11—C16—C15	−0.7 (6)
C1—N1—C9—C8	178.6 (3)	C10—C11—C16—C15	179.8 (4)
C5—C4—C9—N1	−176.7 (3)		

### Hydrogen-bond geometry (Å, °)

**Table:**

Cg is the centroid of the C11–C16 ring.

**Table:**

*D*—H···*A*	*D*—H	H···*A*	*D*···*A*	*D*—H···*A*
O1w—H1w···N1^i^	0.85 (5)	2.02 (5)	2.867 (4)	172 (5)
N2—H2n···O1w^ii^	0.88	2.18	3.007 (4)	157
O1w—H2w···N1	0.85 (5)	2.20 (5)	3.047 (5)	175 (5)
C5—H5···O1w^ii^	0.95	2.45	3.380 (5)	165
C17—H17a···Cg^iii^	0.98	2.65	3.508 (5)	147

Symmetry codes: (i) −*x*+1, −*y*+2, −*z*+1; (ii) −*x*+1, −*y*+1, −*z*+1; (iii) −*x*, −*y*, −*z*+2.
